# Predicting the Future Need of Walking Device or Assistance by Moderate to Vigorous Physical Activity: A 2-Year Prospective Study of Women Aged 75 Years and Above

**DOI:** 10.1155/2018/1340479

**Published:** 2018-06-20

**Authors:** Takuji Adachi, Kuniyasu Kamiya, Yuji Kono, Kotaro Iwatsu, Yuko Shimizu, Ikumi Honda, Sumio Yamada

**Affiliations:** ^1^Program in Physical and Occupational Therapy, Nagoya University Graduate School of Medicine, Nagoya 461-8673, Japan; ^2^Department of Hygiene and Public Health, Osaka Medical College, Takatsuki 569-0801, Japan; ^3^Department of Rehabilitation, Fujita Health University Banbuntane Hotokukai Hospital, Nagoya 454-8509, Japan; ^4^Department of Rehabilitation, Hirakata Kohsai Hospital, Hirakata 573-5103, Japan; ^5^Department of Health Sciences, Nagoya University Graduate School of Medicine, Nagoya 461-8673, Japan

## Abstract

**Objective:**

To examine the association between daily moderate to vigorous physical activity (MVPA) and the change in mobility function among community-dwelling Japanese women aged 75 years and above.

**Methods:**

This prospective study included 330 older women aged 75 years and above who could walk without a walking device or assistance. MVPA and light-intensity physical activity (LPA) were assessed using an accelerometer for seven consecutive days. MVPA was defined as an activity with an intensity of >3 metabolic equivalents. The study outcome was a change in mobility function, defined as the need of walking device or assistance, during the two-year period.

**Results:**

The results of the logistic regression analysis showed that MVPA was inversely associated with a decline in mobility function after controlling for LPA and potential confounders (adjusted odds ratio (OR) = 0.93 per 1 min/d, 95% confidence interval (CI) = 0.88–0.99; *P* = 0.017), whereas LPA was not when adjusted for MVPA and confounders (adjusted OR = 0.99 per 1 min/d, 95% CI = 0.96–1.01; *P* = 0.245). The receiver operating characteristics analysis identified a 7.9 min/d of MVPA as the cut-off value.

**Conclusions:**

The results of this study suggest the importance of promoting daily MVPA for preventing mobility limitation in older women aged 75 years and above.

## 1. Introduction

Declined mobility function results in disability, mortality, and increased healthcare costs in old age [1]. In particular, the early detection and prevention of declining mobility function are important among older women aged 75 years and above because of a higher prevalence of physical frailty compared to younger elderly or men [2]. Additionally, risk of incident of disability is higher in older women compared to men [3].

Physical activity (PA) is a modifiable risk factor associated with the onset of slow gait and disability in old age [4], and the causal relationship of increased PA with reduced risk of mobility limitation has been demonstrated in older people through randomized controlled trial [5]. Therefore, promoting PA is a key intervention to maintain mobility function in old age. However, despite the importance of PA, there is a lack of evidence regarding the relationship between PA and mobility function in older people aged 75 years and above [6].

Moderate to vigorous PA (MVPA), generally defined as an activity with an intensity of >3 metabolic equivalents (METs) such as brisk walking and aerobics [7], has favorable effects on muscle mass [8, 9], falling [10], and mortality [11] in older people. A recent prospective study demonstrated that higher-intensity exercise is associated with fewer disabilities, whereas duration of exercise was not after adjusting for exercise duration [12], suggesting that MVPA may contribute to preserving mobility function in old age. However, this study assessed PA using a questionnaire and also did not consider the influences of potential confounding factors such as muscle strength or depression. Painful joint is another factor that affects PA in older individuals [13]. Therefore, objective measurement of MVPA and adjustment of confounders are needed for evidence regarding the favorable effects of MVPA in old age.

We previously reported the inverse relationship between objectively measured MVPA and slow gait in community-dwelling older women, and a significant relationship was observed even when adjusted for potential confounders [6]. Another recent study on subjects aged 65 years and above also reported that objectively measured moderate-intensity PA is an independent factor of physical performance parameters [14]. However, due to the cross-sectional design, the relationship of MVPA with a change in mobility function was not examined by these studies. Therefore, the present study aimed to examine (1) the relationship between objectively measured MVPA and the future need of walking device or assistance and (2) the cut-off value of MVPA to predict this study outcome in Japanese community-dwelling older women by a prospective design.

## 2. Methods

### 2.1. Study Population and Design

This is a prospective cohort study performed as part of a cohort study conducted in our laboratory at the Graduate School of Medicine, Nagoya University, Japan. The inclusion criterion of the main cohort study was community-dwelling older people aged 75 years and above. Exclusion criterion was those who were not able to access a health checkup at Nagoya University Daiko Campus from their homes. The volunteer participants were recruited from senior citizen's clubs in Nagoya city and registered to the Research of Health Promotion at Nagoya University. The registered participants were invited for a health examination via mail every two years, and all of them provided written informed consent to participate in the study. The present study included older women aged 75 years and above who participated in the health examination in 2012 or 2013 and could walk independently without any walking devices. Participants who were lost to follow-up were also excluded. The study protocol was approved by the Ethics Committee of the School of Health Sciences at Nagoya University (approval number 2012-0131). Two years after the baseline survey was conducted, each participant was further monitored to assess the need for a walking device or assistance in daily living.

### 2.2. Study Outcome

The study outcome was a change in mobility function, which was assessed by follow-up survey of the main cohort study or through telephonic interviews. The study outcome was defined as either the inability to walk without a walking device in daily life or the inability to go out alone because of a health or physical problem. In general, a mobility limitation is defined according to difficulty in walking a certain distance (e.g., 400 m or one-fourth of a mile) or climbing stairs without resting [15]. However, study participants in our study were aged 75 years and above who might have decreased cognitive or executive function. Therefore, we avoided asking the distance that they could walk because it was not likely to be reliable.

### 2.3. Measurement of Physical Activity

To avoid seasonal effects, PA was measured in autumn (September to November) using a uniaxial accelerometer (Kenz Lifecorder, Suzuken Co., Ltd., Nagoya, Japan). The device records step counts and intensity of PA. The intensity of PA was categorized into 11 levels (0, 0.5, 1-9) based on the recorded acceleration pattern.

A previous study that assessed the relationship between the accelerometer levels and METs determined using objectively measured oxygen consumption during walking on a treadmill revealed that an accelerometer level >4 corresponded to >3 METs [16]. Taking this result, PA with an accelerometer level >4 from this device has been widely used to categorize MVPA in both middle-aged and older adults [17–19]. An intensity level of 0 meant no movement and that of 0.5 signified slight body or arm movement, such as deskwork. Therefore, PA with an accelerometer level of 1 to 3 was defined as light-intensity PA (LPA).

Participants were instructed to wear the accelerometers around the waist for seven consecutive days, except during bathing, swimming, and sleeping. Additionally, participants were instructed to continue their normal activities of daily living during the measurement period and were blinded to their PA.

A valid day for analysis was defined as 10 or more h/d of monitor wear [20]. The wear time was determined by subtracting the nonwear time (an interval of at least 20 consecutive minutes of zero activity intensity) from 24 h [20]. The mean durations of MVPA and LPA per day were calculated using five or more valid days [21]. Data from participants with four or less valid days were regarded as missing data.

### 2.4. Demographic Characteristics

Data on age and comorbidities of each participant were collected using a self-administered questionnaire. Body mass index was calculated as body weight (kg) divided by the square of height (m). The number of prescribed medications was counted for each participant by checking their prescriptions. Self-reported pain in the back or leg was also assessed as “none,” “rarely,” “sometimes,” or “always.” Presence of pain for data analysis purposes was defined as feeling pain “sometimes” or “always” with reference to a previous study [13].

### 2.5. Other Covariates

Grip strength was assessed as the representative indicator of muscle strength using a Jamar hydraulic dynamometer (Sammons Preston, USA) set at the second handle position. Grip strength of each participant was measured by trained physical therapist. The participants sat with the wrist in a neutral position and the elbow flexed at 90° [22]. Two trials were completed for each hand and the strongest value was used for the analysis [23]. In our previous study, grip strength was an independent associated factor of slow gait in older women aged 75 years and above [6]. Executive function was assessed using the Trail Making Test (TMT) [24]. TMT is a visual task to connect circles by drawing a line from one point to the next as quickly as possible, in numerical order (TMT-A), and in alternating between both numerical and Japanese-character order (TMT-B). The time to finish TMT-B minus that to finish TMT-A (ΔTMT) was calculated. The ΔTMT score is used for controlling the effect of motor speed on TMT performance and is considered a more accurate measure of executive functions than TMT-B alone [25]. The ΔTMT has also been reported to be correlated with walking performance in Japanese older people [26]. Depression was assessed using the 5-item Geriatric Depression Scale (GDS-5) which is a questionnaire that assesses depression symptoms using five items, and a score of ≥2 points is defined as depression [27]. Depression assessed using GDS-5 was also associated with slow gait in older women [6].

### 2.6. Statistical Analyses

Characteristics of the participants with and without a decline in mobility function were compared by the Mann–Whitney U test or chi-square test. Then logistic regression analysis was performed to examine the relationship between the study outcome and MVPA or LPA. In this analysis, variables that showed a *P*-value of < 0.1 in the univariate analysis were used as independent variables.

In baseline data, missing values were observed in several variables. The logistic regression analysis was first performed only for participants without missing data. Then, to avoid bias associated with excluding missing data, the logistic regression analysis was performed for all participants as a sensitivity analysis. Missing values were imputed using the median for continuous variables and the most frequent category for categorical variables from the available data.

Finally, if MVPA was independently associated with a decline in mobility function, receiver operating characteristic (ROC) curve analysis was conducted to identify the possible cut-off value of MVPA. The ROC curve was constructed by plotting sensitivity against 1-specificity, and cut-off value was selected by optimizing the sensitivity-specificity relationship.

All statistical analyses were performed using the STATA version 14 software package (SPSS Inc., Chicago, IL, USA), and a *P*-value < 0.05 was considered statistically significant.

## 3. Results

**Figure 1:**
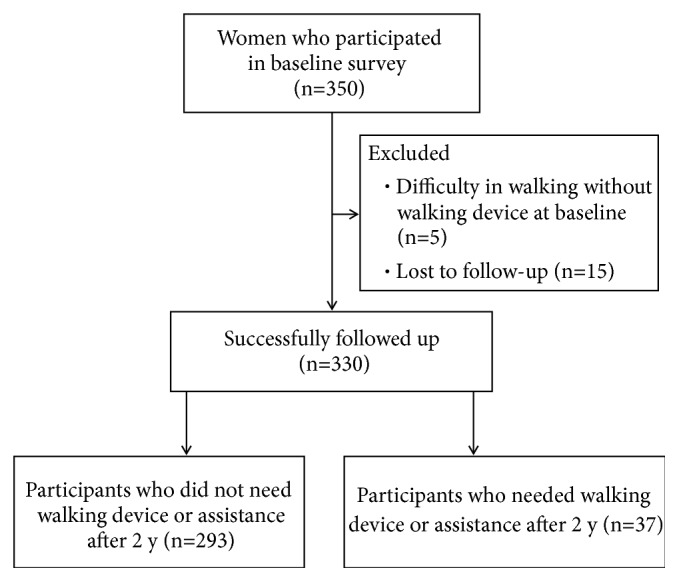
Flow diagram of the study participants.

Of all the 345 older women who could walk without a walking device at baseline, 330 participants who were successfully monitored were enrolled in the analysis (Figure 1). During the two years after the baseline survey, 37 participants (11.2%) newly required walking device or assistance in their daily living. Detailed information about study outcome was shown in Table 1.

Participant characteristics and comparisons of the measured variables between those with and without the study outcome are shown in Table 2. Of the 330 participants analyzed in this study, 300 participants provided PA measurement for five or more valid days (seven days, n = 161; six days, n = 130; five days, n = 9). Participants who required walking device or assistance after two years were older (*P* < 0.001) and used more prescribed medications (*P* = 0.005), had weaker grip strength (*P* < 0.001), and had longer ΔTMT (*P* = 0.002) compared to those who did not experience the study outcome. Participants who experienced the study outcome also showed shorter duration of daily MVPA (*P* < 0.001) and LPA (*P* = 0.002) and tended to have a higher prevalence of depression at baseline survey compared to those without study outcome (*P* = 0.078).

Results of logistic regression analysis using complete data are shown in Table 3. MVPA and LPA were inversely and significantly associated with the need of walking device or assistance after controlling for potential confounding factors (MVPA: adjusted odds ratio (OR) = 0.92 per 1 min/d, 95% confidence interval (CI) = 0.87-0.97;* P* = 0.004, LPA: adjusted OR = 0.97 per 1 min/d, 95% CI = 0.95-0.99;* P* = 0.023). The significant relationship between MVPA and study outcome remained after further adjusting for LPA (adjusted OR = 0.93 per 1 min/d, 95% CI = 0.88-0.99;* P* = 0.017), but LPA was not after adjusting for MVPA (adjusted OR = 0.99 per 1 min/d, 95% CI = 0.96-1.01;* P* = 0.245). The sensitivity analysis, including all participants, also showed the inverse and independent relationship between increased MVPA and study outcome (Table 4).

**Figure 2:**
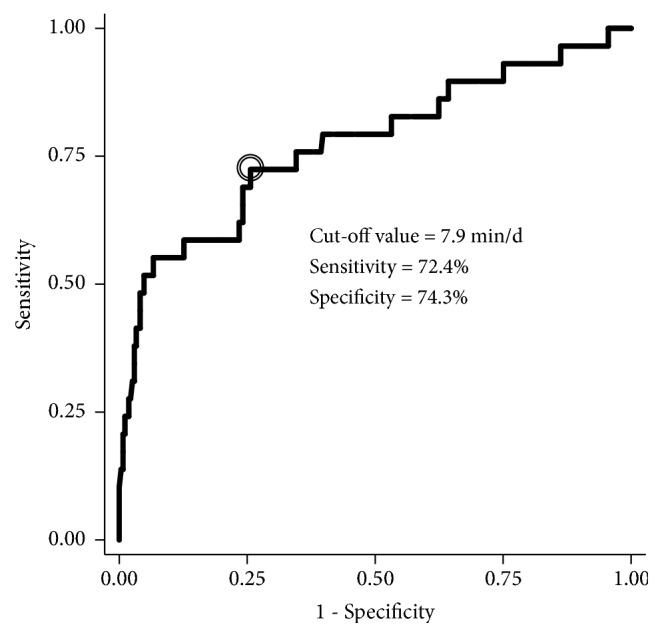
Receiver operating characteristic curve to predict the need of walking device or assistance using moderate to vigorous physical activity. Area under the curve was 0.773 (95% CI: 0.666-0.881).

The ROC curve analysis, using a change in mobility function as an outcome, identified a 7.9 min/d of MVPA as the optimal predictive value, with a sensitivity of 72.4% and a specificity of 74.3%; the AUC was 0.773 (95% CI = 0.666-0.881;* P* < 0.001) (Figure 2).

## 4. Discussion

The main finding of this study was that duration of daily MVPA predicted the change in mobility function, defined as the future need of walking device or assistance, in community-dwelling women aged 75 years and above, even after controlling for potential confounders. Our results suggest that MVPA plays a key role in maintaining mobility function in this population.

In this study, 37 of the 330 participants (11.2%) required the walking device or assistance during the subsequent two years and this study outcome was inversely associated with objectively measured MVPA at baseline. A previous study in older Japanese men and women aged 65 years and above reported that 3.9% of the study participants had incident disability, defined as the care-needs certification in the national long-term care insurance system of Japan, during the two-year period [28]. Although the definitions of study outcome are different, inclusion of older women aged 75 years and above in the present study may result in a relatively high ratio of incident of change in mobility function.

The association between PA in old age and onset of disability has been well documented by observational study [29]. The effects of the intervention based on promoting moderate-intensity PA on reduced risk of mobility limitation have also been shown by a large-scale randomized controlled trial [5]. However, there is little evidence to build robust recommendations for promoting MVPA in older people aged 75 years and above. To this end, we performed a prospective cohort study to examine the relationship of MVPA with a change in mobility function in this population. In our study, daily MVPA was inversely associated with the future need of walking device or assistance after controlling for potential confounding factors. In contrast, LPA was not associated with study outcome, implying the importance of daily MVPA for preserving mobility function.

The ROC curve analysis identified a 7.9 min/d of daily MVPA as a cut-off value for predicting decline in mobility function. This amount is less than 150 min/wk recommended by the World Health Organization [30]; however, the minimum amount of MVPA may differ from population to population. A previous longitudinal study implied that ≥ 15 min/d of MVPA was associated with lower probability of losing muscle mass over 5 years in older people aged 65 years and above [8]. Another cross-sectional study in women aged 60 years and above demonstrated that 107.4 min/d was a cut-off value for predicting mobility limitation [31]. From our results, approximately 60 min/wk may be a target amount of MVPA at which health gains in individuals aged 75 years and above. Further studies are needed to examine the generalizability of our results because of the inclusion of healthier participants in this study.

Increased MVPA resulted in a reduced risk of the need of walking device or assistance due to several possible mechanisms. First, daily MVPA may maintain mobility function through preserving muscle mass and strength that declines with aging and becomes a determinant of mobility function [32]. A previous cohort study reported that objectively measured MVPA had a negative correlation with loss of lean body mass in older people [8]. A more recent cohort study showed that a higher amount of self-reported MVPA was related to reduce risk of sarcopenia [9]. The effect of PA on peripheral nerve function is another possible mechanism. It has been reported that long-term moderate- to high-intensity brisk walking improves peripheral nerve function in diabetic patients [33]. Considering the relationship between peripheral nerve function and walking ability [34], daily MVPA is likely to affect mobility function via peripheral nerve function.

Furthermore, a lack of social participation due to physical inactivity has a possibility of playing a role in declined mobility function. A previous longitudinal study reported that social participation outside the home was inversely related to onset and progression of disability [35]. Because moderate-intensity activity but not light intensity is related to life-space mobility that refers to the area a person moves through in daily life in older individuals [36], decreased MVPA could lead to a negative cycle of reduced life-space mobility and social participation and in turn to decline in mobility function.

The study has several limitations that should be discussed. First, the study participants voluntarily participated in the main cohort study. Added to this, they were recruited at senior citizen's clubs, potentially causing selection bias. This may limit the generalization of our findings to frailer or physically disabled participants. As noted before, generalizability should be examined by further study. Second, although the participants were blind to their PA during the measurement period and were instructed to continue their normal activity, wearing accelerometer might have stimulated their PA. Hence, the amount of walking activity might be overestimated, possibly affecting the cut-off value of MVPA identified by the ROC analysis. Third, there may be other unknown confounding factors such as level of education or living circumstance. Finally, the causal relationship should be confirmed by an interventional study. Nevertheless, the present study has clinical significance in terms of implying a preventive effect of MVPA on change in mobility function in women aged 75 years and above.

## 5. Conclusions

In this prospective study, we examined the association between daily MVPA and change in mobility function among community-dwelling older women aged 75 years and above. The results showed that objectively measured MVPA predicted the future need of walking device or assistance, independent of LPA and other potential confounders. In contrast, LPA was not related to the study outcome when controlling for MVPA and other confounding factors. Our results indicate that 60 min/wk can be appropriate amount of MVPA for the target population due to the small sample size.

## Figures and Tables

**Table 1:** Detail information about study outcome.

	Number of participants who newly required walking device or assistance after 2 years	
	In a house	Outside
Need of walking device	16	28
Need of assistance for walking	10	20

**Table 2:** Characteristics of the study participants and comparison of characteristics of those with and without the study outcome.

Variables	Overall (N = 330)	n	Participants who did not need walking device and assistance (n = 293)	n	Participants who newly required walking device or assistance (n = 37)	n	*P*
Age, years	79 [77-82]	330	79 [77-82]	293	83 [78-85]	37	<0.001
BMI, kg/m^2^	21.6 [19.6-23.9]	330	21.6 [19.7-24.0]	293	21.6 [18.3-23.2]	37	0.243
Hypertension, n (%)	168 (50.9)	330	149 (45.2)	293	19 (51.4)	37	0.938
Diabetes, n (%)	47 (14.2)	330	41 (14.0)	293	6 (16.2)	37	0.685
Dyslipidemia, n (%)	147 (44.5)	330	135 (46.1)	293	12 (32.4)	37	0.118
Stroke, n (%)	14 (4.2)	330	12 (4.1)	293	2 (5.4)	37	0.669
Heart disease, n (%)	28 (8.5)	330	23 (7.8)	293	5 (13.5)	37	0.235
Prescribed medication, number	1 [1-4]	330	1 [1-4]	293	4 [1-6]	37	0.005
Pain, n (%)	83 (25.2)	330	70 (24.0)	293	13 (35.1)	37	0.141
Grip strength, kg	20 [18-22]	327	20 [18-23]	291	18 [16-20]	36	<0.001
ΔTMT, sec	78.0 [57.0-125.7]	327	75.8 [54.2-117.1]	291	126.3 [74.5-188.2]	36	0.002
Depression, n (%)	55 (16.7)	328	45 (15.3)	291	10 (27.0)	37	0.078
MVPA	13.8 [6.3-24.0]	300	14.6 [7.7-24.7]	269	2.1 [0.5-10.7]	31	<0.001
LPA	50.5 [39.1-63.4]	300	51.2 [40.3-63.9]	269	39.3 [22.6-51.7]	31	0.002

Continuous variables are shown by median [interquartile range]. BMI, body mass index; TMT, Trail Making Test; MVPA, moderate to vigorous physical activity; and LPA, light-intensity physical activity.

**Table 3:** Results of the logistic regression analysis in participants with complete data (n=300).

	Model 1			Model 2		Model 3			
	OR	95% CI	*P*	OR	95% CI	*P*	OR	95% CI	*P*
Age, per 1 year	1.16	[1.03-1.31]	0.016	1.15	[1.03-1.30]	0.014	1.14	[1.01-1.29]	0.032
Prescribed medications, per 1 medication	1.09	[0.95-1.26]	0.213	1.12	[0.98-1.28]	0.089	1.09	[0.95-1.25]	0.197
Grip strength, per 1 kg	0.93	[0.82-1.06]	0.279	0.91	[0.80-1.03]	0.141	0.91	[0.81-1.05]	0.213
ΔTMT, per 1 sec	1.00	[0.99-1.01]	0.107	1.00	[0.99-1.01]	0.189	1.00	[0.99-1.01]	0.153
Depression, yes	1.55	[0.56-4.25]	0.397	1.75	[0.65-4.73]	0.271	1.56	[0.56-4.31]	0.390
MVPA, per 1 min/d	0.92	[0.87-0.97]	0.004	-			0.93	[0.88-0.99]	0.017
LPA, per 1 min/d	-			0.97	[0.95-0.99]	0.023	0.99	[0.96-1.01]	0.245

Dependent variable: need of walking device or assistance; OR, odds ratio; CI, confidence interval; TMT, Trail Making Test; MVPA, moderate to vigorous physical activity; and LPA, light-intensity physical activity.

**Table 4:** Results of the logistic regression analysis including all participants (n = 330).

	Model 1			Model 2			Model 3		
	OR	95% CI	*P*	OR	95% CI	*P*	OR	95% CI	*P*
Age, per 1 year	1.13	[1.02-1.27]	0.018	1.14	[1.02-1.26]	0.016	1.13	[1.01-1.25]	0.032
Prescribed medications, per 1 medication	1.09	[0.97-1.23]	0.136	1.12	[0.99-1.26]	0.053	1.10	[0.97-1.24]	0.126
Grip strength, per 1 kg	0.91	[0.81-1.01]	0.081	0.89	[0.79-0.99]	0.041	0.89	[0.80-1.00]	0.059
ΔTMT, per 1 sec	1.01	[1.00-1.01]	0.011	1.00	[1.00-1.01]	0.026	1.01	[1.00-1.01]	0.017
Depression, yes	1.33	[0.54-3.26]	0.534	1.43	[0.59-3.47]	0.431	1.33	[0.54-3.26]	0.535
MVPA, per 1 min/d	0.93	[0.89-0.98]	0.004	-			0.94	[0.90-0.99]	0.017
LPA, per 1 min/d	-			0.97	[0.95-0.99]	0.030	0.99	[0.96-1.01]	0.255

Dependent variable: need of walking device or assistance; OR, odds ratio; CI, confidence interval; TMT, Trail Making Test; MVPA, moderate to vigorous physical activity; and LPA, light-intensity physical activity.

## Data Availability

All relevant data are within the paper.
